# Neck Circumference as a Practical Anthropometric Biomarker for Visceral Adiposity and Metabolic Dysregulation in Type 2 Diabetes

**DOI:** 10.3390/metabo16020093

**Published:** 2026-01-26

**Authors:** Meixia Ji, Zhifu Zeng, Zhengliang Huang, Zhaowei Shi, Meifen Ji

**Affiliations:** 1 Lishui People’s Hospital, Lishui 323000, China; zmjmx0416@163.com (M.J.); zengzhifu2025@163.com (Z.Z.); 18957091397@163.com (Z.S.); 2 Disease Prevention and Control Center of Jingning She Autonomous County, Lishui 323500, China; hzljmx12688@163.com; 3 Department of Nursing, The First Affiliated Hospital of Zhejiang University School of Medicine, Hangzhou 310003, China

**Keywords:** neck circumference, biomarker, visceral fat, type 2 diabetes, insulin resistance, public health, prevention, metabolic syndrome

## Abstract

Objective: Visceral adipose tissue is a primary driver of insulin resistance and dysglycemia in type 2 diabetes (T2D), yet its clinical assessment remains challenging. This study aimed to validate neck circumference (NC) as a novel, practical anthropometric biomarker for estimating visceral fat area (VFA) and identifying metabolic risk in a T2D cohort, facilitating its integration into public health and primary care screening strategies. Methods: In a cross-sectional study of 1139 T2D patients, we collected data on NC, biochemical parameters (fasting plasma glucose, hemoglobin A1c, high-density lipoprotein cholesterol, low-density lipoprotein cholesterol, triglycerides), and precisely measured VFA and subcutaneous fat area (SFA) via bioelectrical impedance analysis (Omron HDS-2000). We employed Pearson’s correlation and multivariate logistic regression to analyze the relationship between NC and metabolic indicators. Receiver operating characteristic (ROC) curve analysis was used to establish sex-specific NC cut-off values for predicting abnormal VFA. Results: The cohort comprised 687 (60.3%) males and 452 (39.7%) females. NC demonstrated strong positive correlations with VFA (*p* < 0.001), as did body mass index (BMI), waist–hip ratio (WHR), and SFA. In males, NC was further positively correlated with key metabolic biomarkers including fasting insulin, Insulin Resistance Index, triglycerides, and creatinine. ROC analysis identified NC > 39.5 cm for males and >35.5 cm for females as the optimal cut-off points for detecting abnormal visceral adiposity, highlighting its diagnostic utility. Conclusions: NC serves as a highly accessible and effective biomarker for visceral adiposity and associated metabolic dysfunction in patients with T2D. The established sex-specific cut-off values provide a simple, non-invasive tool for risk stratification in clinical and public health settings, enabling early intervention and improved management of metabolic disease.

## 1. Introduction

In a 2021 release of the Global Diabetes Map, the International Diabetes Federation (IDF) reported that the global number of adults with diabetes had reached 537 million [1]. In recent years, China has witnessed a continuous increase in the number of diabetes cases, attributable to improvements in living standards, changes in lifestyle habits, and an aging population. A 2025 research report from the Chinese Center for Disease Control and Prevention indicates that the age-standardized overall prevalence of diabetes in China (including children and adolescents, without distinguishing between diabetes types) was 13.7% in 2023, affecting approximately 233 million individuals—an increase of 163% compared to 2005. If current trends continue unabated, the prevalence of diabetes is projected to rise linearly, reaching 16.15% by 2030, 21.52% by 2040, and 29.10% by 2050 [2]. However, the treatment and control rates for diabetes in China remain relatively low, with both the rates of treatment and attainment of target levels being less than 50% among diagnosed patients. Poor long-term glucose control can lead to chronic complications such as neuropathy, vascular disorders, retinopathy, and nephropathy. These complications not only severely affect the quality of life of patients but also impose significant economic burdens on them. The 2021 IDF Global Diabetes Map estimated that diabetes-induced global health expenditure in 2021 amounted to approximately 966 billion USD, accounting for 9% of global health expenditure [1]. Acute and chronic complications, including diabetic ketoacidosis, uremia, and severe diabetic foot, can be life-threatening. In 2021, an estimated 6.7 million adults died from diabetes or its complications, comprising 12.2% of global mortalities [1]. While the national awareness and control rate of diabetes have improved, there are still regional disparities, and the control of diabetes in rural areas remains unoptimistic, especially in remote rural areas. The above data show that the prevention and control of diabetes has become a challenge that our country and even the world need to face. Therefore, as medical professionals, early screening and prevention of diabetes is an important task.

Patients with T2D often have obesity and insulin resistance as comorbidities, and obesity is itself a significant independent risk factor for T2D [3]. In obesity, adipose tissue undergoes pathological remodeling characterized by adipocyte hypertrophy, altered adipokine secretion, cell death, hypoxia, and impaired stromal vascular function. This remodeling triggers chronic, low-grade inflammation driven by immune cell infiltration and dysregulation. The resulting inflammatory state promotes systemic insulin resistance, thereby increasing the risk of T2D [4]. Studies have shown that individuals with abdominal obesity (especially those with increased visceral fat) are at a higher risk of developing diabetes and metabolic syndrome than those with peripheral obesity. Some studies have reported that visceral fat area (VFA) more strongly influences fasting plasma glucose (FPG), total cholesterol (TC), triglycerides (TG), and uric acid levels than subcutaneous fat area (SFA). Clinically, a subset of obese patients exhibit normal metabolic parameters, a condition termed metabolically healthy obesity (MHO); these patients often predominantly have peripheral obesity. Kang et al. used bioelectrical impedance to measure VFA in participants; their results showed that VFA is associated with metabolic indicators such as body mass index (BMI), waist-to-hip ratio (WHR), FPG, TC, TG, and the prevalence of hypertension and diabetes [5]. Therefore, accurate assessment of VFA is crucial for the early diagnosis and risk stratification of patients with T2D, enabling early intervention and improved prognosis.

Methods for measuring VFA often include precise instrument measurement and human parameter measurement. Currently recommended techniques for evaluation of fat distribution commonly use devices such as computed tomography (CT) and MRI; dual-energy X-ray absorptiometry can directly assess visceral fat content and accurately evaluate total fat and distribution; the new bioimpedance method has a good correlation with current MRI measurement of VFA [6]. However, these instrument-based methods have relatively high technical requirements and associated economic costs. Additionally, CT and dual-energy X-ray examinations expose individuals to radiation, making them unsuitable for large-scale population screening. In clinical practice, anthropometric measurements such as BMI, waist circumference (WC), and WHR are commonly used to assess obesity. However, BMI cannot distinguish between fat and muscle mass. In contrast, WC and WHR are better predictors of abdominal obesity. These measures offer the advantages of simplicity and low cost and correlate well with the risk of metabolic syndrome and cardiovascular diseases. However, the accuracy of these measurements can be influenced by factors such as clothing in colder seasons and recent food or water intake, leading to significant variability when measured at different times. Therefore, there is a need to identify simpler, accurate, and more economical indicators for assessing visceral adiposity.

NC (neck circumference), measured at the level of the thyroid cartilage (Adam’s apple), reflects upper-body fat distribution. This measurement is convenient, cost-effective, and highly reproducible. Its accuracy is not influenced by clothing or dietary status, making it an increasingly favored tool in clinical research. In a study of 123 patients with snoring and suspected sleep apnea, Kazmi et al. first identified NC and obesity as significant predictors of sleep apnea [7]. A study by Li et al. involving 177 Chinese adults demonstrated a positive correlation between NC and VFA [8]. Data from the Framingham Heart Study (*n* = 3307) established a correlation between NC and cardiovascular disease risk factors [9]. However, there are few studies on the correlation between NC and VFA in the Chinese diabetic population. This study utilized bioelectrical impedance analysis (BIA) to measure VFA in patients with T2D. We aimed to investigate the correlations between NC and VFA, along with other metabolic parameters. Furthermore, we evaluated the potential of NC as a predictive indicator for visceral adiposity and its utility as a screening tool for abnormal VFA in this population. We propose that NC could offer a reliable, convenient, and cost-effective method for estimating visceral fat content in clinical practice. This could facilitate earlier intervention and improve management strategies for T2D.

## 2. Materials and Methods

### 2.1. Research Subjects

#### 2.1.1. Inclusion Criteria

(1) Hospitalized patients aged 18 to 75 years with a confirmed diagnosis of T2D according to the 1999 World Health Organization diagnostic criteria. (2) Patients admitted to the Department of Endocrinology, Lishui People’s Hospital, between April 2018 and February 2020. (3) Willing and able to provide informed consent for participation in the study.

#### 2.1.2. Exclusion Criteria

(1) Presence of concurrent severe hepatic or renal failure, or acute diabetic complications; (2) Presence of neck masses; thyroid enlargement suggested by ultrasonography or on palpation; history of corticosteroid use; or history of Cushing’s syndrome, pituitary, or adrenal diseases; (3) Pregnancy; (4) History of neck surgery.

The study protocol was approved by the Institutional Ethics Committee of Lishui People’s Hospital (The Sixth Affiliated Hospital of Wenzhou Medical University) (Approval No.: 2022-19). The study was conducted in full compliance with the principles of the Declaration of Helsinki and relevant local regulations. Written informed consent was obtained from all participants prior to their enrollment.

### 2.2. Basic Data

The following data were collected for all participants: (1) Demographics and anthropometrics: gender, age, occupation, educational level, annual income, height, weight, NC, WC, and hip circumference (HC). (2) Biochemical parameters: FPG, fasting insulin (FINS), 2-h plasma glucose (2hPG), 2-h plasma insulin (2h-PI), hemoglobin A1c (HbA1c), high-density lipoprotein cholesterol (HDL-c), low-density lipoprotein cholesterol (LDL-C), TG, and TC. (As detailed in Table S1).

Based on these measurements, BMI and WHR were calculated. Visceral and subcutaneous fat areas were also determined. All data were collected by researchers who had undergone standardized training.

### 2.3. Testing Methods

#### Calculation Formulas

(1) Insulin Resistance Index (HOMA-IR): FPG (mmol/L) × FINS (μU/mL)/22.5. (2) BMI: Weight (kg)/Height^2^ (m^2^). (3) WHR: WC/HC.

### 2.4. Diagnostic Criteria

#### 2.4.1. Underweight

BMI < 18.5 kg/m^2^; Normal Weight: 18.5 kg/m^2^ ≤ BMI < 24 kg/m^2^; Overweight: 24 kg/m^2^ ≤ BMI < 28 kg/m^2^; Obese: BMI ≥ 28 kg/m^2^.

#### 2.4.2. Visceral Fat Grading

Abnormal VFA (Central Obesity): VFA ≥ 100 cm^2^; Normal VFA: VFA < 100 cm^2^.

### 2.5. Statistical Analysis

Data were analyzed with SPSS (version 17.0; IBM Corp., Armonk, NY, USA) for general statistics and with R (version 3.2.3; R Foundation for Statistical Computing, Vienna, Austria) for locally weighted scatterplot smoothing (LOWESS). *t*-tests and variance analysis were used to compare NC differences among patients with diabetes under different conditions; Pearson’s correlation analysis was used to study the correlation between body parameters, metabolic indices, and NC; multivariate logistic regression analysis was used to analyze risk factors for abnormal VFA; LOWESS was used to analyze the linear trend between NC and VFA; SPSS software was used to draw ROC curves and evaluate the cut-off point for diagnosing abnormal VFA by NC. Measurement data were represented as mean ± standard deviation, and were analyzed by *t*-test/variance analysis; count data were represented as rates (%), and were analyzed by chi-square test. The test level α = 0.05. *p* < 0.05 indicated statistical significance.

## 3. Results

### 3.1. General Characteristics and Testing Indicators of the Research Subjects

#### 3.1.1. General Characteristics of Diabetes Patients

The study enrolled 1139 participants, comprising 687 (60.3%) males and 452 (39.7%) females. The majority were aged 40–59 years, with a mean age of 53.64 ± 10.28 years. Among male participants, 42% had attained a high school education or higher, compared to 12.6% of females. Occupational distribution was relatively balanced among males, whereas the majority of females were homemakers or unemployed (60.0%). Household income was predominantly distributed across the 31,000–100,000 and 101,000–300,000 ranges, as shown in Table 1.

#### 3.1.2. Physical Indicators, Physiological and Biochemical Testing Indicators of the Study Population

The NC of the participants ranged from 29.0 to 50.0 cm, with an overall mean of 37.28 ± 3.41 cm. Values were higher in males (38.85 ± 2.84 cm) than in females (34.89 ± 2.71 cm). The VFA ranged from 5.0 to 247.0 cm^2^, with an overall mean of 78.94 ± 37.89 cm^2^. Males exhibited a higher VFA (81.89 ± 41.13 cm^2^) compared to females (74.47 ± 31.90 cm^2^). As shown in Table 2, except for BMI, all other physical measures differed significantly between genders (*p* < 0.05). Specifically, males had significantly greater NC, WHR, and VFA than females (*p* < 0.05). In contrast, the age and systolic blood pressure of females were higher than males (*p* < 0.05). Table 3 presents further metabolic comparisons between genders. Except for 2hPG, HOMA-IR, TC, and LDL-C, all other biochemical indices showed statistically significant differences between males and females (*p* < 0.05).

### 3.2. The Association Between Neck Circumference and Visceral Fat

#### Correlation of Neck Circumference with Body Parameters and Metabolic Indicators

NC exhibited statistically significant differences (*p* < 0.05) across subgroups defined by age, gender, educational attainment, and income level among patients with T2D. Neck circumference exhibits a decreasing trend with age. The highest mean value was observed in participants aged 18–39 years. Participants with a high school education or less had a larger NC (38.34 ± 3.30 cm) than those with more than a high school education (36.80 ± 3.33 cm). NC increased with BMI, reaching 40.42 ± 3.22 cm in the obese group. Additionally, patients with an abnormal VFA showed a significantly greater NC (39.88 ± 3.13 cm) compared to those with a normal VFA (36.33 ± 2.97 cm) (*p* < 0.001). These results are summarized in Table 4.

Pearson correlation analysis revealed that NC was significantly and positively correlated with BMI, WHR, VFA, and SFA in both genders (all *p* < 0.001), as shown in Table 5.

Significant correlations were identified between NC and key metabolic parameters. For the total cohort, positive correlations were found with FPG, 2hPG, HOMA-IR, TG, and creatinine, while an inverse correlation was observed with HDL-c. Gender-stratified analysis indicated that in males, NC was positively associated with FINS, 2h-PI, HOMA-IR, TG, TC, and creatinine, and negatively associated with HDL-c (all *p* < 0.05). In females, the only significant correlation was with HDL-c; all other associations were non-significant (*p* > 0.05). The complete data are shown in Table 6.

### 3.3. Linear Relationship Between Neck Circumference and Visceral Fat Area in the Study Population

**Figure 1:**
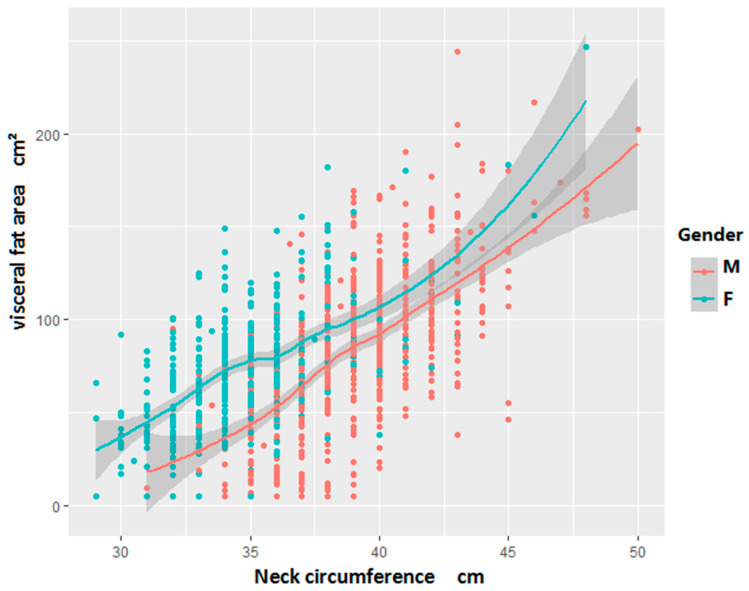
Local weighted regression scatter plot of neck circumference and VFA (Visceral Fat Area) in the study population by different genders.

The relationship between NC and VFA was analyzed using the LOWESS method. The analysis revealed a positive linear association, indicating that VFA increased with NC, as illustrated in Figure 1.

### 3.4. Best Cut-Off Value for Neck Circumference Related to Central Obesity Diagnosis

**Figure 2:**
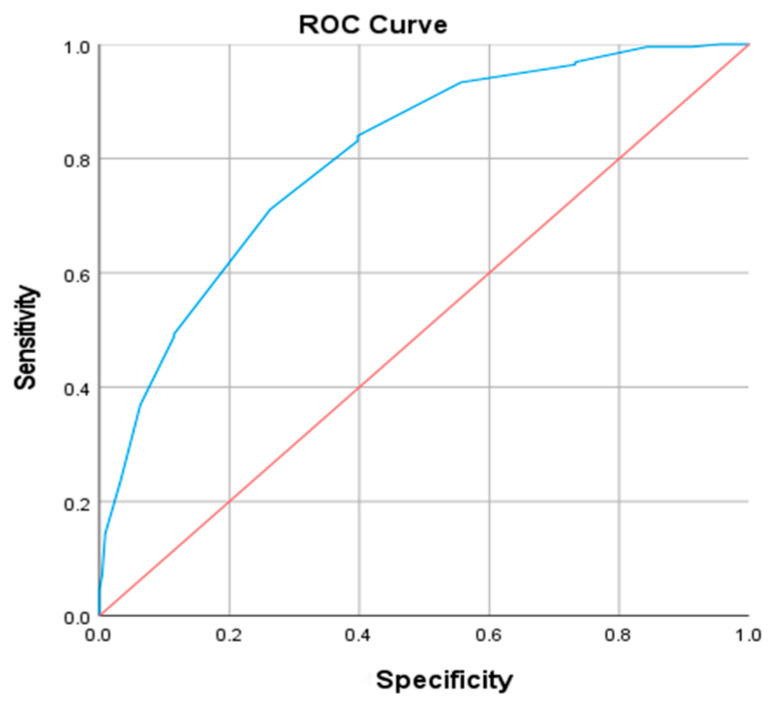
ROC curve of neck circumference diagnosis of abnormal visceral fat area in male patients. The blue line represents the ROC curve of NC, the red line indicates the reference line (AUC = 0.5).

**Figure 3:**
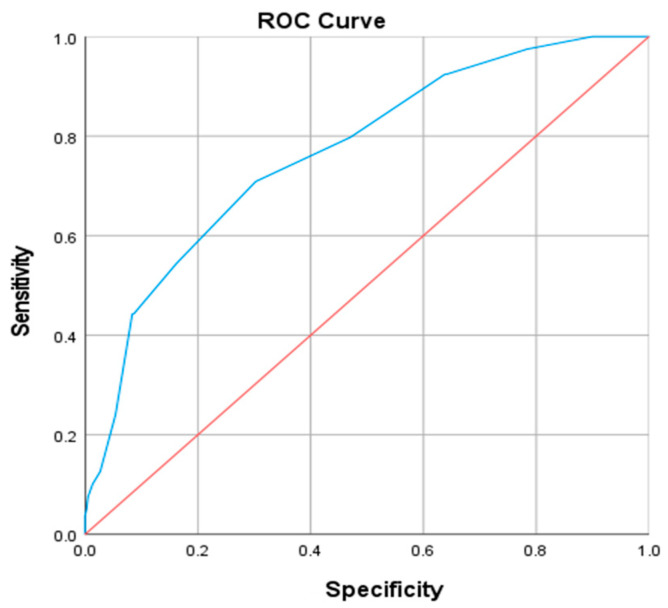
ROC curve of neck circumference diagnosis of abnormal visceral fat area in female patients. The blue line represents the ROC curve of NC, the red line indicates the reference line (AUC = 0.5).

Receiver operating characteristic (ROC) curve analysis was performed to assess the predictive value of NC for VFA abnormality (defined as >100 cm^2^). For males, the area under the curve (AUC) was 0.802 (*p* < 0.001). At the optimal cut-off point of 39.5 cm (maximizing the Youden index), sensitivity was 0.711 and specificity was 0.736 (Figure 2). Similarly, in females, the AUC was 0.764 (*p* < 0.001), with a cut-off value of 35.5 cm yielding a sensitivity of 0.709 and a specificity of 0.692 (Figure 3).

### 3.5. Multivariate Logistic Regression Analysis of Visceral Fat Area Abnormality

#### 3.5.1. Univariate Analysis

Univariate analysis using chi-square tests for categorical variables and *t*-tests for continuous variables identified several factors significantly associated with VFA abnormality. These included sex, hypertension, WHR, BMI, NC, 2h-PI, TG, TC, creatinine, and HDL-c (all *p* < 0.05), as detailed in Table 7.

#### 3.5.2. Multivariate Logistic Regression Analysis

The relationship between metabolic indicators and VFA was assessed using stepwise multivariate logistic regression. The outcome variable (VFA) was dichotomized into abnormal (Y = 1, ≥100 cm^2^) and normal (Y = 0, <100 cm^2^). The initial independent variables included sex, hypertension, WHR, BMI, NC, 2h-PI, TG, TC, creatinine, and HDL-c. Following adjustment and variable selection, NC, TC, BMI, and WHR were retained in the final model. The analysis confirmed that increased values of these variables were independently associated with a greater risk of abnormal VFA (Table 8).

## 4. Discussion

Obesity, an escalating global health challenge, exerts detrimental effects on multiple organ systems and significantly compromises quality of life. The rising incidence of T2D is largely attributable to modifiable risk factors, including the global obesity epidemic, physical inactivity, and unhealthy dietary patterns [10]. This progressive rise in obesity prevalence poses substantial economic burdens on healthcare systems and societies worldwide. The World Health Organization ranks obesity as the fifth leading risk factor for global mortality. Obesity can be categorized based on the distribution of fat into peripheral and central obesity. In peripheral obesity, subcutaneous fat primarily accumulates in the buttocks and lower limbs, while central obesity manifests as an increase in visceral adipose tissue and ectopic fat deposition. Central obesity is more strongly associated with insulin resistance and T2D than peripheral obesity [11,12]. This distinction is underscored by a study of 43 sedentary postmenopausal women by Brochu et al., which reported that metabolically healthy obese individuals possessed 49.6% less visceral adipose tissue than their metabolically abnormal counterparts (141 ± 53 cm^2^ vs. 211 ± 85 cm^2^; *p* < 0.01) [13]. This is likely due to the stronger lipolytic activity in visceral fat cells compared to peripheral fat, which leads to the overproduction of free fatty acids, the generation of toxic metabolites in the liver, and subsequent impairment of cellular function and production of inflammatory factors. This also affects insulin signaling pathways, leading to insulin resistance and abnormalities in glucose and lipid metabolism, making individuals with central obesity more susceptible to metabolic diseases.

Currently, several medical imaging techniques are available for assessing visceral obesity, including dual-energy X-ray absorptiometry (DEXA), CT, MRI [14], and bioelectrical impedance analysis. However, the high cost, limited accessibility, and radiation exposure associated with DEXA, CT, and MRI restrict their utility for large-scale population screening. Consequently, there is a compelling need for simple, reliable anthropometric surrogate measures to identify individuals with elevated visceral adiposity during routine health examinations. Commonly, BMI is the most widely used indicator to measure total fat, and WC, WHR, and WHtR have been used as alternative indicators for visceral fat. In our study, NC in the total population was positively correlated with BMI and WHR. After distinguishing by gender, NC in both men and women was still positively correlated with BMI and WHR, which is consistent with previous research findings [15,16]. A cross-sectional study on elderly Chinese participants found a high correlation between NC and BMI and WC [17]. Another study on diabetic patients found that NC is positively correlated with central obesity and metabolic syndrome [18]. Research by Okosun [19] and others also indicates a correlation between NC and overweight and obesity, and it shows good correlation with weight, WC, hip circumference, BMI, and WHR in both men and women. However, BMI cannot distinguish between body fat and muscle tissue, nor identify the anatomical location or function of different fat depots. WC has many limitations, the measurement site for WC varies among different clinical studies [20,21], and WC measurements can be influenced by stomach fullness and respiration, making it impractical for large-scale population studies, especially in cold weather and while wearing heavy clothing. The accumulation of fat around the neck is a unique phenomenon that describes subcutaneous fat tissue in the upper body. NC has been proposed as an alternative indicator of upper body subcutaneous fat distribution and has been proven to be closely associated with other anthropometric parameters (e.g., BMI and WC) and various metabolic risk factors [15,16,22,23]. Owing to its well-defined anatomical landmarks, minimal variability with respiration or meals, high reproducibility, and low cost, NC is considered a superior anthropometric indicator for assessing central obesity compared to traditional measures [24]. Thus, NC represents a simple, effective, and reliable tool for identifying central obesity in clinical and public health settings.

In the present study, NC demonstrated significant metabolic correlations in the overall population, showing positive associations with FBG and 2hPG, creatinine, HOMA-IR, and TG, and a negative correlation with HDL. Gender-stratified analysis revealed that in males, NC remained positively correlated with FINS, 2h-PI, HOMA-IR, creatinine, TC, and TG, while inversely correlated with HDL-c (all *p* < 0.05). In females, however, NC was significantly associated only with HDL-c, with no statistically significant correlations observed with other metabolic indicators such as HbA1c, FPG, TG, or TC (*p* > 0.05). According to studies by Hoebel et al. [25] and others [26,27], NC can be used as an effective evaluation index of risk factors for metabolic syndrome, such as HOMA-IR, central obesity, blood pressure, FPG, and TG. Further reinforcing its clinical relevance, NC has also been linked to cardiometabolic risk factors in adolescent populations [27]. Notably, evidence suggests that NC may serve as an independent risk factor for metabolic syndrome, potentially offering superior predictive value compared to conventional measures such as BMI, WC, and WHR [28]. The association between neck fat and metabolic syndrome and its components may be attributed to the excessive release of free fatty acids from the upper body subcutaneous fat to plasma [9], which, in turn, has high contents of free fatty acids associated with oxidative stress and markers of insulin resistance [29], which in turn affect blood sugar.

The emergence of visceral fat can be explained as a specific marker of systemic lipid overaccumulation, manifested by an increase in circulating TG levels. Excess lipids may be stored in ectopic sites (such as skeletal muscle, liver, and pancreatic β cells), where they cause substantial metabolic disruption [14]. Furthermore, visceral fat can produce more free fatty acids [14] and secrete a large amount of inflammatory cytokines, cell, and fat factors, which may play a critical role in the generation of insulin resistance and the onset of diabetes [30]. Yang et al. [31] reported that, among 18 severely obese (BMI > 40 kg/m^2^), non-diabetic individuals, NC significantly correlated with both VFA (r^2^ = 0.67, *p* < 0.0001) and HOMA-IR (r^2^ = 0.35, *p* = 0.01). In the same cohort, WC was associated with VFA (r^2^ = 0.25, *p* = 0.03) but not with HOMA-IR. These measurements were derived from anthropometry, single-slice CT analysis at L4, and fasting blood samples. In our study, Pearson correlation analysis demonstrated a significant positive correlation between NC and VFA in the total population (r = 0.566, *p* < 0.001), with a weaker but still significant correlation with HOMA-IR (r = 0.088, *p* = 0.02). The association with VFA remained consistent in both gender subgroups. This finding reinforces the view that NC is a more robust indicator of visceral adiposity than other anthropometric parameters. Hong-xing Li et al. [8] found a significant correlation between NC and VFA in their analysis of CT scans from 177 Chinese patients. They attribute this correlation to a significant association between abdominal fat area and neck fat area. LiZhao et al. [32] reported, in a cohort of 9366 individuals, that NC was independently associated with visceral obesity. After comprehensive adjustment for confounders, linear regression confirmed a significant positive correlation between NC and visceral obesity indices in both sexes (all *p* < 0.001), with risk increasing progressively across NC quartiles. Our findings are consistent with this pattern. We observed that NC increased with BMI, reaching 40.42 ± 3.22 cm in the obese group. Furthermore, NC was significantly higher in individuals with an abnormal VFA compared to those with a normal VFA (*p* < 0.001). This positive association was corroborated by LOWESS analysis, which revealed a clear trend of increasing VFA with larger NC in both genders. Importantly, multivariate logistic regression analysis confirmed that NC remained independently associated with visceral obesity after adjustment for sex and hypertension.

The threshold of NC associated with overweight or obesity differs among studies. According to a study by Yang et al. [18], the NC cut-off points for predicting central obesity and overweight are 35 cm for females and 37 cm and 38 cm for males. In another study [33], the cut-off points for diagnosing central obesity were 37.1 cm for males (with sensitivity of 0.767 and specificity of 0.741), and 32.6 cm for females (with sensitivity of 0.833 and specificity of 0.878). When considering the entire population, the cut-off point for diagnosing central obesity was 36.1 cm (with sensitivity of 0.741 and specificity of 0.735). To predict over-weight, the cut-off points were 37.4 cm for males (with sensitivity of 0.709 and specificity of 0.763), and 32.2 cm for females (with sensitivity of 0.783 and specificity of 0.853). For the entire population, the cut-off point to predict overweight was 37.5 cm (with sensitivity of 0.486 and specificity of 0.873). These differences might be due to variations in the research population and diagnostic criteria, among other factors. There is currently a paucity of studies using NC to predict VFA in diabetic populations. In our study, ROC curve analysis demonstrated that NC exhibited significant predictive power for abnormal VFA in both genders. The area under the curve (AUC) was 0.802 (*p* < 0.001) for males, with an optimal cut-off value of 39.5 cm. For females, the AUC was 0.764 (*p* < 0.001), with an optimal cut-off of 35.5 cm. A Shanghai-based study involving 2477 men and 3107 women indicated that relying solely on BMI, WC, WHR, or WHtR may be suboptimal for predicting metabolic risk factors and related chronic diseases in Chinese adults [34]. In contrast, NC offers advantages of greater practicality, reliability, and higher reproducibility compared to BMI and WHR. Future research should explore the incorporation of NC alongside traditional anthropometric indices to enhance early screening strategies for metabolic disorders, an approach with considerable potential for improving clinical practice and preventive care.

The advantage of our study is that all the individuals are from the same ethnicity, eliminating racial influence. Human body measurements and questionnaire surveys were conducted by a unified, trained research team, maintaining strict quality control. However, there were also shortcomings. For instance, our participants were T2D patients in clinical research, and our results have yet to be verified in community settings and the general population. Our study lacked direct measurement of sub-cutaneous and visceral fat, such as CT and MRI, which could accurately reflect fat distribution. It should be recognized that diet habits, diet control, and other factors of T2D patients could have a significant impact on NC. Some physically active individuals may have larger NC, and these factors were not eliminated in our study. Furthermore, relevant research shows a correlation between NC and sleep apnea syndrome [35]. Thyroid function parameters were not measured in our study and could introduce some error into our results; future studies including thyroid hormone assessment may further clarify its role in the relationship between NC and metabolic risk. Despite these limitations, we were still able to hypothesize on the utility of NC in predicting VFA. Future research should expand the sample size, study different patient groups, include healthy population comparisons, and consider additional research factors like sleep habits, snoring, exercise, diet habits, etc., to better establish the correlation between NC, metabolic indicators, and VFA. Additionally, the repeatability of the results of the NC cut-off point for diagnosing abnormal VFA should be further researched.

## 5. Conclusions

This study demonstrates that neck circumference (NC) is significantly associated with visceral fat area (VFA) and various metabolic parameters in patients with type 2 diabetes. Specifically, NC showed a strong positive correlation with VFA, BMI, and waist–hip ratio. Furthermore, we identified sex-specific cut-off values (NC > 39.5 cm for males and NC > 35.5 cm for females) for predicting abnormal VFA (≥100 cm^2^) with good sensitivity and specificity. These findings suggest that NC, as a simple and non-invasive anthropometric measure, could serve as a practical and cost-effective screening tool for assessing visceral adiposity and associated cardiometabolic risk in clinical settings. Future prospective studies are warranted to validate these cut-off values and establish their utility in risk stratification and intervention strategies.

## Figures and Tables

**Table 1:** Basic demographic characteristics of the study population (n, %).

Classification	Male (*n* = 687)	Female (*n* = 452)	Total
Age (year)			
18–39	90 (13.1)	19 (4.2)	109 (9.6)
40–59	419 (61.0)	246 (54.4)	665 (58.4)
60–75	178 (25.9)	187 (41.4)	365 (32.0)
Education			
High school and above	294 (42.8)	57 (12.6)	351 (30.8)
Below High School	393 (57.2)	395 (87.4)	788 (69.2)
Career			
Public officials/Personnel of enterprises and institutions	205 (29.8)	76 (16.8)	281 (24.7)
Business and service personnel	140 (20.4)	38 (8.4)	178 (15.6)
Professional skill worker/Clerk	52 (7.6)	10 (2.2)	62 (5.4)
Houseworker/Unemployed	161 (23.4)	271 (60.0)	432 (37.9)
Other workers	129 (18.8)	57 (12.6)	186 (16.3)
Annual household income (yuan)			
30,000 and below 30,000 yuan	41 (6.0)	61 (13.5)	102 (9.0)
31,000 to 100,000 yuan	249 (36.2)	221 (48.9)	470 (41.3)
110,000 to 300,000 yuan	379 (55.2)	163 (36.1)	542 (47.6)
Over 300,000 yuan	18 (2.6)	7 (1.5)	25 (2.2)

**Table 2:** Results of physical and physiological indicators in the study population by different genders (±s).

Classification	Male (*n* = 687)	Female (*n* = 452)	Total
Age (year) ^#^	51.53 ± 10.65	56.85 ± 8.77	53.64 ± 10.28
Neck circumference (cm) ^#^	38.85 ± 2.84	34.89 ± 2.71	37.28 ± 3.41
Waist-to hip ratio (%) ^#^	95.37 ± 6.53	93.39 ± 6.93	94.59 ± 6.76
Diastolic blood pressure (mmHg) ^#^	77.42 ± 10.65	74.39 ± 9.69	76.22 ± 10.39
Systolic blood pressure (mmHg) ^#^	128.31 ± 17.17	132.63 ± 18.77	130.02 ± 17.94
body mass index (kg/m^2^)	25.21 ± 3.23	24.65 ± 3.53	24.99 ± 3.36
Visceral Fat Area (cm^2^) *	81.89 ± 41.13	74.47 ± 31.9	78.94 ± 37.89

*: *p* < 0.05; ^#^: *p* < 0.001.

**Table 3:** Results of biochemical tests in the study population by different genders (±s).

Classification	Male (*n* = 687)	Female (*n* = 452)	Total
Hemoglobin A1c (%) *	10.21 ± 2.44	9.67 ± 2.25	9.99 ± 2.38
Fasting plasma glucose (mmol/L) ^#^	10.28 ± 3.57	9.48 ± 3.27	9.96 ± 3.48
2 h plasma Glucose (mmol/L)	17.87 ± 5.44	17.25 ± 4.86	17.62 ± 5.22
Fasting insulin (uU/mL) ^#^	10.08 ± 11.33	13.70 ± 26.50	11.47 ± 18.73
2 h plasma insulin (uU/mL) ^#^	36.77 ± 48.92	52.02 ± 63.86	42.63 ± 55.60
Insulin resistance index	4.52 ± 5.52	5.61 ± 10.96	4.94 ± 8.06
Blood urea nitrogen (mmol/L) ^#^	5.54 ± 1.5 7	5.08 ± 1.66	5.35 ± 1.62
Serum creatinine (μmol/L) ^#^	71.15 ± 20.77	55.14 ± 17.69	64.8 ± 21.11
Triglycerides (mmol/L) ^#^	2.72 ± 3.02	2.17 ± 1.7	2.5 ± 2.59
Total cholesterol (mmol/L)	5.02 ± 1.5	5.06 ± 1.25	5.03 ± 1.41
High density lipoprotein (mmol/L) ^#^	1.13 ± 0.43	1.25 ± 0.37	1.18 ± 0.41
Low density lipoprotein (mmol/L)	2.57 ± 0.85	2.62 ± 0.8	2.59 ± 0.83

*: *p* < 0.05; ^#^: *p* < 0.001.

**Table 4:** Comparison of neck circumference in the study population.

Classification	Number of Cases	Neck Circumference (cm)	*t*/*F* Value	*p* Value
Age (year)			29.21	<0.001
18–39	109	38.93 ± 3.32		
40–59	665	37.51 ± 3.39		
60–73	365	36.36 ± 3.17		
Gender			23.37 *	<0.001
Male	687	38.85 ± 2.84		
Female	452	34.89 ± 2.71		
Education			−7.23 *	<0.001
High school and above	788	36.80 ± 3.33		
below High School	351	38.34 ± 3.30		
Annual household income (yuan)			18.30	<0.001
Over 300,000 yuan	25	38.64 ± 4.20		
110,000 to 300,000 yuan	542	37.93 ± 3.33		
31,000 to 100,000 yuan	470	36.74 ± 3.26		
30,000 and below	102	35.91 ± 3.33		
Hypertension			1.97 *	0.047
NO	773	37.14 ± 3.31		
YES	363	37.57 ± 3.57		
BMI			190.63	<0.001
<18.5	23	32.35 ± 2.69		
18.5~23.9	410	35.23 ± 2.57		
24.0~27.9	517	37.97 ± 2.75		
≥28.0	189	40.42 ± 3.22		
Visceral fat area			17.58 *	<0.001
normal	835	36.33 ± 2.97		
abnormal	304	39.88 ± 3.13		

*: *t* value.

**Table 5:** Correlation analysis (Pearson) between neck circumference, body parameters, and visceral fat area in the study population by different genders.

Classification	Male (*n* = 687)		Female (*n* = 452)		Total Population	
	*r* Value	*p* Value	*r* Value	*p* Value	*r* Value	*p* Value
BMI	0.777	<0.001	0.692	<0.001	0.653	<0.001
Waist-to hip ratio (%)	0.437	<0.001	0.343	<0.001	0.406	<0.001
Visceral fat area	0.647	<0.001	0.588	<0.001	0.566	<0.001
Subcutaneous fat area	0.71	<0.001	0.567	<0.001	0.492	<0.001

**Table 6:** Correlation analysis (Pearson) between neck circumference and metabolic indicators in the study population by different genders.

Classification	Male (*n* = 687)		Female (*n* = 452)		Total Population	
	*r* Value	*p* Value	*r* Value	*p* Value	*r* Value	*p* Value
Hemoglobin A1c (%)	−0.061	0.109	−0.055	0.242	0.015	0.624
Fasting plasma glucose (mmol/L)	0.054	0.158	0.027	0.56	0.101	0.001
2 h plasma glucose (mmol/L)	0.055	0.152	0.021	0.663	0.068	0.022
Fasting insulin (uU/mL)	0.275	<0.001	0.081	0.185	0.066	0.082
2 h plasma insulin (uU/mL)	0.196	<0.001	0.087	0.156	0.042	0.271
HOMA-IR	0.272	<0.001	0.075	0.220	0.088	0.020
Serum creatinine (μmol/L)	0.08	0.036	0.031	0.508	0.260	<0.001
Triglycerides (mmol/L)	0.231	<0.001	0.036	0.444	0.203	<0.001
Total cholesterol (mmol/L)	0.127	0.001	−0.09	0.06	0.036	0.227
High density lipoprotein (mmol/L)	−0.183	<0.001	−0.151	0.001	−0.218	<0.001
Low density lipoprotein (mmol/L)	0.037	0.329	−0.05	0.323	−0.013	0.655

**Table 7:** Results of 2-value test for categorical data and *t*-test for continuous data.

Variable	cχ^2^ (*t*) Value	*p* Value
Age	−1.301	0.193
Gender	32.503	<0.001
Career	8.143	0.086
Annual household income	3.615	0.306
Education	2.241	0.134
Hypertension	14.094	<0.001
Waist-to hip ratio (%)	15.160	<0.001
Neck circumference	17.575	<0.001
BMI	21.920	<0.001
Hemoglobin A1c (%)	−1.553	0.121
Fasting plasma glucose (mmol/L)	1.891	0.059
2 h plasma glucose (mmol/L)	1.185	0.236
Fasting insulin (uU/mL)	1.633	0.103
2 h plasma insulin (uU/mL)	2.701	0.007
Serum creatinine (μmol/L)	3.793	<0.001
Blood urea nitrogen (mmol/L)	1.285	0.199
Triglycerides (mmol/L)	4.838	<0.001
Total cholesterol (mmol/L)	2.716	0.007
High density lipoprotein (mmol/L)	−3.530	<0.001
Low density lipoprotein (mmol/L)	0.4701	0.639

**Table 8:** Multifactor logistic regression analysis of factors influencing abnormal visceral fat area.

Variable	β	Standard Error (Sx)	Wald χ^2^ Value	Degree of Freedom	*p* Value	OR Value	95% CI
BMI	0.402	0.049	67.487	1	0	1.495	1.358~1.645
Waist to hip ratio	0.096	0.018	28.584	1	0	1.101	1.063~1.141
Neck-circumference	0.169	0.038	19.711	1	0	1.184	1.099~1.276
Total cholesterol	0.139	0.068	4.193	1	0.041	1.15	1.006~1.314
Constant	−28.441	2.285	154.953	1	0	0	

## Data Availability

The data presented in this study are available on request from the corresponding author.

## Supplementary Materials

The following supporting information can be downloaded at: https://www.mdpi.com/article/10.3390/metabo16020093/s1, Table S1: Detection of Anthropometric Parameters and Metabolic Indices.

## References

[1] Magliano D.J., Boyko E.J., IDF Diabetes Atlas 10th Edition Scientific Comittee (2021). IDF DIABETES ATLAS.

[2] Zhou Y., Liu J., Zhao Z., Zhou M., Ng M. (2025). The national and provincial prevalence and non-fatal burdens of diabetes in China from 2005 to 2023 with projections of prevalence to 2050. Mil. Med. Res..

[3] American Diabetes Association Professional Practice Committee (2024). 2. Diagnosis and Classification of Diabetes: Standards of Care in Diabetes-2024. Diabetes Care.

[4] Choe S.S., Huh J.Y., Hwang I.J., Kim J.I., Kim J.B. (2016). Adipose Tissue Remodeling: Its Role in Energy Metabolism and Metabolic Disorders. Front. Endocrinol..

[5] Kang S.H., Cho K.H., Park J.W., Yoon K.W., Do J.Y. (2015). Association of visceral fat area with chronic kidney disease and metabolic syndrome risk in the general population: Analysis using multi-frequency bioimpedance. Kidney Blood Press. Res..

[6] Browning L.M., Mugridge O., Chatfield M.D., Dixon A.K., Aitken S.W., Joubert I., Prentice A.M., Jebb S.A. (2010). Validity of a new abdominal bioelectrical impedance device to measure abdominal and visceral fat: Comparison with MRI. Obesity.

[7] Katz I., Stradling J., Slutsky A.S., Zamel N., Hoffstein V. (1990). Do patients with obstructive sleep apnea have thick necks?. Am. Rev. Respir. Dis..

[8] Li H.-X., Zhang F., Zhao D., Xin Z., Guo S.-Q., Wang S.-M., Zhang J.-J., Wang J., Li Y., Yang G.-R. (2014). Neck circumference as a measure of neck fat and abdominal visceral fat in Chinese adults. BMC Public Health.

[9] Preis S.R., Massaro J.M., Hoffmann U., D’Agostino R.B., Levy D., Robins S.J., Meigs J.B., Vasan R.S., O’Donnell C.J., Fox C.S. (2010). Neck circumference as a novel measure of cardiometabolic risk: The Framingham Heart study. J. Clin. Endocrinol. Metab..

[10] Ershow A.G. (2009). Environmental influences on development of type 2 diabetes and obesity: Challenges in personalizing prevention and management. J. Diabetes Sci. Technol..

[11] Vague J. (1956). The degree of masculine differentiation of obesities: A factor determining predisposition to diabetes, atherosclerosis, gout, and uric calculous disease. Am. J. Clin. Nutr..

[12] Peppa M., Koliaki C., Papaefstathiou A., Garoflos E., Katsilambros N., Raptis S.A., Hadjidakis D.I., Dimitriadis G.D. (2013). Body composition determinants of metabolic phenotypes of obesity in nonobese and obese postmenopausal women. Obesity.

[13] Brochu M., Tchernof A., Dionne I.J., Sites C.K., Eltabbakh G.H., Sims E.A., Poehlman E.T. (2001). What are the physical characteristics associated with a normal metabolic profile despite a high level of obesity in postmenopausal women?. J. Clin. Endocrinol. Metab..

[14] Zhang M., Zheng L., Li P., Zhu Y., Chang H., Wang X., Liu W., Zhang Y., Huang G. (2016). 4-Year Trajectory of Visceral Adiposity Index in the Development of Type 2 Diabetes: A Prospective Cohort Study. Ann. Nutr. Metab..

[15] Luo Y., Ma X., Shen Y., Xu Y., Xiong Q., Zhang X., Xiao Y., Bao Y., Jia W. (2017). Neck circumference as an effective measure for identifying cardio-metabolic syndrome: A comparison with waist circumference. Endocrine.

[16] Dai Y., Wan X., Li X., Jin E., Li X. (2016). Neck circumference and future cardiovascular events in a high-risk population—A prospective cohort study. Lipids Health Dis..

[17] Yan Q., Sun D., Li X., Zheng Q., Li L., Gu C., Feng B. (2014). Neck circumference is a valuable tool for identifying metabolic syndrome and obesity in Chinese elder subjects: A community-based study. Diabetes Metab. Res. Rev..

[18] Yang G.-R., Yuan S.-Y., Fu H.-J., Wan G., Zhu L.-X., Bu X.-L., Zhang J.-D., Du X.-P., Li Y.-L., Ji Y. (2010). Neck circumference positively related with central obesity, overweight, and metabolic syndrome in Chinese subjects with type 2 diabetes: Beijing Community Diabetes Study 4. Diabetes Care.

[19] Okosun I.S., Liao Y., Rotimi C.N., Choi S., Cooper R.S. (2000). Predictive values of waist circumference for dyslipidemia, type 2 diabetes and hypertension in overweight White, Black, and Hispanic American adults. J. Clin. Epidemiol..

[20] Millar S.R., Perry I.J., Van den Broeck J., Phillips C.M. (2015). Optimal central obesity measurement site for assessing cardiometabolic and type 2 diabetes risk in middle-aged adults. PLoS ONE.

[21] Stabe C., Vasques A.C.J., Lima M.M.O., Tambascia M.A., Pareja J.C., Yamanaka A., Geloneze B. (2013). Neck circumference as a simple tool for identifying the metabolic syndrome and insulin resistance: Results from the Brazilian Metabolic Syndrome Study. Clin. Endocrinol..

[22] Hingorjo M.R., Qureshi M.A., Mehdi A. (2012). Neck circumference as a useful marker of obesity: A comparison with body mass index and waist circumference. J. Pak. Med. Assoc..

[23] Li Q., Wang N., Han B., Chen Y., Zhu C., Chen Y., Xia F., Cang Z., Zhu C., Chen C. (2015). Neck circumference as an independent indicator to non-alcoholic fatty liver disease in non-obese men. Nutr. Metab..

[24] Hingorjo M.R., Zehra S., Imran E., Qureshi M.A. (2016). Neck circumference: A supplemental tool for the diagnosis of metabolic syndrome. J. Pak. Med. Assoc..

[25] Hoebel S., Malan L., de Ridder J.H. (2012). Determining cut-off values for neck circumference as a measure of the metabolic syndrome amongst a South African cohort: The SABPA study. Endocrine.

[26] He F., He H., Liu W., Lin J., Chen B., Lin Y., Zhao Y., Tao W., Xia X. (2017). Neck circumference might predict gestational diabetes mellitus in Han Chinese women: A nested case-control study. J. Diabetes Investig..

[27] da Silva C.d.C., Zambon M.P., Vasques A.C.J., Rodrigues A.M.d.B., Camilo D.F., Antonio M.Â.R.d.G.M., Cassani R.S.L., Geloneze B. (2014). Neck circumference as a new anthropometric indicator for prediction of insulin resistance and components of metabolic syndrome in adolescents: Brazilian Metabolic Syndrome Study. Rev. Paul. Pediatr..

[28] Zhou J.-Y., Ge H., Zhu M.-F., Wang L.-J., Chen L., Tan Y.-Z., Chen Y.-M., Zhu H.-L. (2013). Neck circumference as an independent predictive contributor to cardio-metabolic syndrome. Cardiovasc. Diabetol..

[29] Santosa S., Jensen M.D. (2008). Why are we shaped differently, and why does it matter?. Am. J. Physiol. Endocrinol. Metab..

[30] Al-Daghri N.M., Al-Attas O.S., Alokail M.S., Alkharfy K.M., Charalampidis P., Livadas S., Kollias A., Sabico S.L., Chrousos G.P. (2013). Visceral adiposity index is highly associated with adiponectin values and glycaemic disturbances. Eur. J. Clin. Investig..

[31] Yang L., Samarasinghe Y.P., Kane P., Amiel S.A., Aylwin S.J.B. (2010). Visceral adiposity is closely correlated with neck circumference and represents a significant indicator of insulin resistance in WHO grade III obesity. Clin. Endocrinol..

[32] Zhao L., Huang G., Xia F., Li Q., Han B., Chen Y., Chen C., Lin D., Wang N., Lu Y. (2018). Neck circumference as an independent indicator of visceral obesity in a Chinese population. Lipids Health Dis..

[33] Pei X., Liu L., Imam M.U., Lu M., Chen Y., Sun P., Guo Y., Xu Y., Ping Z., Fu X. (2018). Neck circumference may be a valuable tool for screening individuals with obesity: Findings from a young Chinese population and a meta-analysis. BMC Public Health.

[34] Gu J.J., Rafalson L., Zhao G.M., Wu H.Y., Zhou Y., Jiang Q.W., Bai Y., Zhu Q.L., Fu X.J., Zhang H. (2011). Anthropometric measurements for prediction of metabolic risk among Chinese adults in Pudong new area of Shanghai. Exp. Clin. Endocrinol. Diabetes.

[35] Chang E.-T., Yang M.-C., Wang H.-M., Lai H.-L. (2014). Snoring in a sitting position and neck circumference are predictors of sleep apnea in Chinese patients. Sleep Breath..

