# Supplementary material for: Neck Circumference as a Practical Anthropometric Biomarker for Visceral Adiposity and Metabolic Dysregulation in Type 2 Diabetes
Source: Metabolites. 2026 Jan 26;16(2):93. doi: 10.3390/metabo16020093 (PMC12943593; doi:10.3390/metabo16020093)
Supplement: Supplementary file 1 [file metabolites-16-00093-s001.zip › metabolites-4049519-supplementary.pdf]

Table S1. Detection of Anthropometric Parameters and Metabolic Indices

Test items	Test method
Waist circumference、Hip circumference、height、weight	The patient wakes up in the morning on an empty stomach, empties and defecates, wears thin clothes, and breathes calmly and lightly. Special personnel take the halfway between the lower ribs and the iliac crest to measure the waist circumference; hip circumference is measured at the largest circumference around the buttocks; Body weight and height were measured using a combined digital scale and stadiometer (Omron, Shenzhen, China).
Neck circumference	The patient standing upright and facing forwards, with shoulders relaxed, the neck circumference was measured below the larynx (Adam's Apple) and perpendicular to the long axis of the neck.
Blood pressure	Rest at least 5 minutes before the measurement; avoid emotional excitement, use an electronic sphygmomanometer (Omron, Dalian, China) to measure: systolic blood pressure (SBP) and diastolic blood pressure (DBP).
Visceral fat area、Subcutaneous fat area	The patients were fasting and supine, breathing calmly, visceral fat area and Subcutaneous fat area was measured by the Omron visceral detection device HDS-2000 (Omron Healthcare Co., Ltd., Kyoto, Japan).
Blood index	After fasting for 10 hours, venous blood samples were collected the next morning. Serum was separated, and biochemical parameters, including FPG, 2hPG, SCr, BUN, HDL-C, LDL-C, TG, and TC, were measured using an automatic biochemical analyzer (Autobio, Zhengzhou, China) with its proprietary reagents. FINS and 2hINS levels were measured on a Cobas e analyzer (Roche Diagnostics, Basel, Switzerland). HbA1c was measured using a Mindray analyzer (Shenzhen Mindray Bio-Medical Electronics Co., Ltd., Shenzhen, China)
